# Zeaxanthin and Other Carotenoids: Roles in Abiotic Stress Defense with Implications for Biotic Defense

**DOI:** 10.3390/plants14172703

**Published:** 2025-08-30

**Authors:** Barbara Demmig-Adams, Amy K. Hodges, Stephanie K. Polutchko, William W. Adams

**Affiliations:** Department of Ecology and Evolutionary Biology, University of Colorado, Boulder, CO 80309, USA; amy.hodges@colorado.edu (A.K.H.); stephanie.polutchko@colorado.edu (S.K.P.);

**Keywords:** abscisic acid, hydrogen peroxide, jasmonic acid, salicylic acid, singlet oxygen, ROS, zeaxanthin

## Abstract

Xanthophylls are carotenoids with diverse roles in stress protection across all taxa of life. This review highlights chloroplast-localized xanthophylls (with a focus on zeaxanthin) of plants by presenting an overview of the protective effects of xanthophylls as well as the role of carotenoids as precursors of multiple plant stress hormones. It also examines the roles of xanthophylls and stress hormones in signaling cascades between the chloroplast and nuclear genes that control plant growth, development, and stress defenses. This overview addresses the biosynthetic pathways of xanthophylls and carotenoid-derived plant stress hormones, functions of xanthophylls in photoprotection of photosynthesis, carotenoids as essential human micronutrients, and roles of xanthophylls in membrane integrity. Attention is given to the involvement of zeaxanthin in both abiotic and biotic defense as well as its impact on components of the biotic defense system with contrasting targets. Examples for the multiple principal loops of signaling cascades between the chloroplast and nucleus, which are based on chloroplast redox state and modulated by xanthophylls, are summarized. This review integrates the role of chloroplast carotenoids in controlling light-use efficiency and providing photoprotection with their system-wide regulatory effects as precursors of carotenoid-derived plant stress hormones and modulators of chloroplast redox state. A better understanding of these connections is needed to guide development of plant lines with improved resilience and productivity in complex, changing, and challenging environments.

## 1. Introduction

Xanthophylls are a class of yellow pigments with diverse roles across all taxa of life, ranging from extremophile microorganisms to humans [1]. This review focuses primarily on multiple supportive roles of xanthophylls synthesized in plant chloroplasts and other plastids, while highlighting similarities to relevant roles in other organisms.

This summary tracks developments in the field over time, focusing on the role of zeaxanthin and similar xanthophylls in modulating the efficiency with which light is used in photosynthesis and their interconversions in xanthophyll cycles [2]. This review also offers insights into (i) roles of carotenoids as biosynthetic precursors of multiple plant stress hormones synthesized in the chloroplast and (ii) roles of both xanthophylls and stress hormones in signaling cascades between chloroplast and nucleus, which regulate carotenoid levels, stress hormone levels, and a myriad of other stress responses, as well as plant growth and development in response to environmental cues perceived and processed in the chloroplast. This review provides a general overview of insights available from different sub-disciplines spanning photoprotection of photosynthesis, phytohormone biology, abiotic stress defense, biotic stress defense, redox signaling, and plant–microbe interaction. A focus of the review is the role of zeaxanthin in abiotic stress protection and gene regulation, with interactions also emerging between zeaxanthin and biotic defense. These findings are discussed in the context of dynamic natural environments and future efforts to develop plants with enhanced stress resilience while avoiding unintended costs. This review does not aim to present an in-depth account of each of the many components and wide-ranging roles of redox signaling, nor the many roles of plant stress hormones, and instead refers to authoritative reviews as appropriate. The review is focused on the role of xanthophylls (especially zeaxanthin) as metabolites that provide direct stress protection and modulate retrograde (organelle-to-nucleus) signals originating in the chloroplast. The latter impacts large networks of nuclear-encoded genes, which parallels recent emphasis on the role of chloroplast antioxidant enzymes, such as ascorbate peroxidase, in retrograde signaling [3]. These roles of carotenoids are also placed in the context of similarly concurrent roles of dietary carotenoids in direct stress protection as well as broad-ranging gene modulation in humans. Lastly, this review touches on emerging evidence that microorganisms target zeaxanthin synthesis in the plant chloroplast to manipulate suites of downstream gene targets.

## 2. Biosynthetic Pathways of Xanthophylls and Carotenoid-Derived Plant Stress Hormones

**Figure 1:**
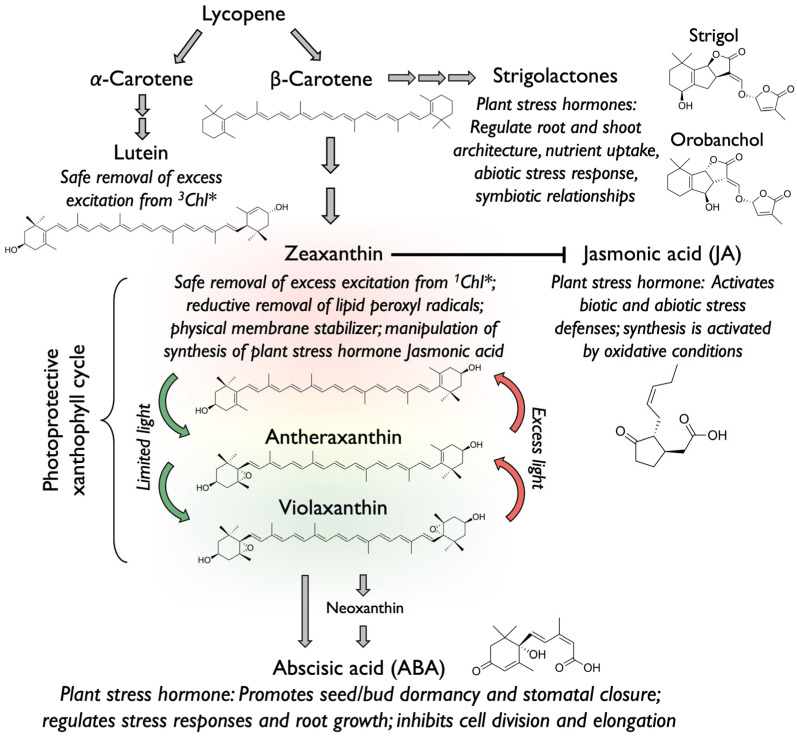
Overview of carotenoid-synthesis pathways, roles of the carotenoids formed, and connections to the biosynthesis of carotenoid-derived plant stress hormones. This schematic illustrates the metabolic conversion of carotenoids (e.g., lycopene, α-carotene, β-carotene) to key xanthophylls and stress hormones in plants. Arrows indicate the flow of these pathways and how different compounds are related. ^1^Chl*, singlet excited chlorophyll; ^3^Chl*, triplet excited chlorophyll.

Figure 1 summarizes information from diverse sub-disciplines that is used below as a basis to discuss emerging roles of xanthophylls in not only abiotic but also biotic defense. The biosynthetic pathway of xanthophylls starts with the carotene (tetraterpene) lycopene as the precursor of both α-carotene and β-carotene, which give rise to the xanthophylls (oxygen-containing carotenoids) lutein and zeaxanthin, respectively (Figure 1). In plants, lutein and zeaxanthin have well-studied roles in the photoprotection of chlorophyll and photosynthesis [1,4]. Furthermore, Figure 1 depicts connections between carotenoid biosynthesis and the biosynthesis of plant stress hormones that are carotenoid-derived and/or impacted by the actions of chloroplast carotenoids in modulating the level of reactive oxygen species (ROS), as is the case for zeaxanthin and jasmonic acid. Overall, the carotenoid pathway in plants is stimulated under stress [5], especially the production of xanthophylls, including the interconvertible components of the xanthophyll cycle (Figure 1). Plants grown in high-light versus low-light environments or those grown under the influence of other environmental stressors feature higher ratios of carotenoids to chlorophyll with a particularly pronounced larger pool of the xanthophylls zeaxanthin + antheraxanthin + violaxanthin and, to a lesser extent, lutein content [2].

In this section, the role of carotenoids as precursors for several plant stress hormones is highlighted with a brief definition of these hormones’ roles (Figure 1). It should be noted that these hormones all have multiple, diverse roles not only in stress defense but also in plant growth and development [6]. β-carotene is the precursor in the synthesis of strigolactones (Figure 1), a class of plant stress hormones that act via local and long-distance communication [7] and contribute to regulating root and shoot architecture, nutrient uptake, abiotic stress response, and symbiotic relationships [8]. Strigolactones are synthesized in a pathway that converts all-trans-β-carotene to 9-cis-β-carotene and then (via cleavage by carotenoid cleavage dioxygenase 7) to 9-cis-β-apo-carotenal, which is further converted (by carotenoid cleavage dioxygenase) to 9-cis-β-apo-10′-carotenal and then carlactone, which is converted to strigolactone in the cytosol [9].

α-Carotene is the precursor of the xanthophyll lutein that functions in photoprotective modulation of chloroplast ROS levels (Figure 1; [10]). Conversely, β-Carotene is the precursor of the xanthophyll zeaxanthin, which indirectly impacts the synthesis of the stress hormone jasmonic acid (JA; Figure 1; [11]). These links between zeaxanthin and jasmonic acid, and possible trade-offs between zeaxanthin’s roles in abiotic and biotic defense as well as between different types of biotic defenses, are a key focus of this review.

Zeaxanthin is also a biosynthetic precursor of violaxanthin, which, in turn, is a precursor (with the xanthophyll neoxanthin as the intermediate of an indirect pathway) of the plant stress hormone abscisic acid (ABA; Figure 1; [12]). ABA is a plant stress hormone that promotes seed/bud dormancy and stomatal closure, regulates stress responses and root growth, and can inhibit cell division and elongation. 9-cis-epoxycarotenoid dioxygenases (NCEDs) cleave the 9-cis-epoxycarotenoids violaxanthin or neoxanthin to form the ABA precursor xanthoxin. Gene expression of nuclear-encoded chloroplast NCED3 is upregulated by H_2_O_2_ signals [13]. ABA travels long-distance from shoots to roots in the phloem (as well as from roots to shoots in the xylem; [14]).

## 3. Photoprotection of Photosynthesis by Xanthophylls

**Figure 2:**
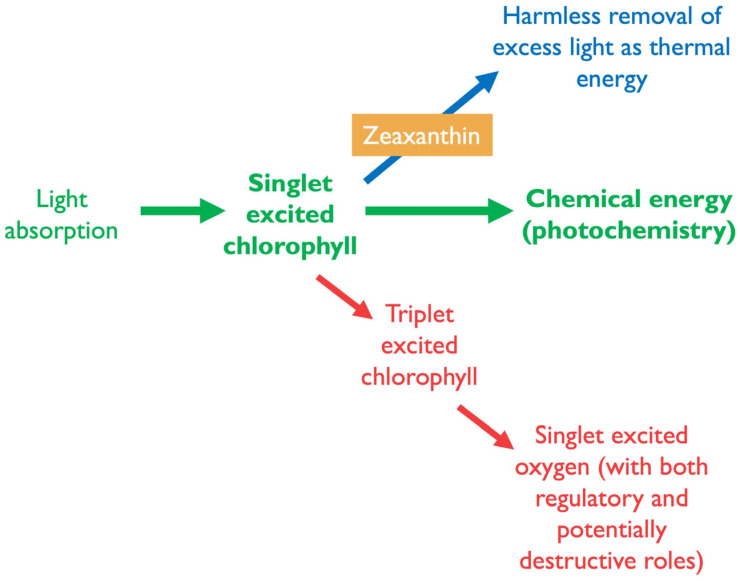
Schematic depiction of the major pathways of energy allocation following light absorption by chlorophyll. Upon light absorption, chlorophyll enters an excited singlet state. This energy can be used in photochemistry to generate chemical energy (green; middle arrow) or can follow either of two alternative pathways (blue, top arrow, and red, bottom arrow). Zeaxanthin plays a key photoprotective role by facilitating the harmless dissipation of excess light energy.

Zeaxanthin protects photosynthesis and the leaf whenever more light is absorbed than can be consumed by photochemistry [2]. Specifically, zeaxanthin facilitates de-excitation of excess singlet state chlorophyll (^1^Chl*; Figure 1; [15]) when the capacity of photochemistry is insufficient to fully utilize this excitation energy either in high-light environments or in the presence of additional environmental stressors [2]. The resulting excess absorbed light can become destructive if substantial energy is passed on to oxygen, leading to the formation of large amounts of ROS. This includes the ROS singlet excited oxygen (^1^O_2_*) formed via energy transfer from singlet excited (^1^Chl*) to triplet excited chlorophyll (^3^Chl*) and then to O_2_ (Figure 2) in the reaction center of photosystem II, as well as from chlorophyll molecules in the light-collecting antenna [16]. Furthermore, another ROS, superoxide (singly reduced oxygen, a radical anion, O_2_^•−^), can be formed when excess energy is passed into the photosynthetic electron transport chain, leading to its over-reduction and transfer of single electrons to oxygen from photosystem I as well as other sites [17,18]. By de-exciting excess ^1^Chl*, zeaxanthin also counteracts over-reduction of the electron transport chain and superoxide production [19].

Zeaxanthin thus protects against excess ROS formation by proactively removing energy (via thermal dissipation) from the same excited state of chlorophyll (^1^Chl*) that passes energy into photosynthesis (Figure 1; [2,20]). This function could interfere with energy utilization in photosynthesis under limiting light availability. However, the level of zeaxanthin is under tight control from a regulatory system that only permits zeaxanthin formation in the xanthophyll cycle (Figure 1), and engagement in thermal energy dissipation, when excess light is present. Upon return to non-excessive light, zeaxanthin is typically swiftly removed by conversion to antheraxanthin and violaxanthin, completing the xanthophyll cycle (Figure 1; [2,20]). Moreover, plants experiencing higher levels of light stress also exhibit larger total xanthophyll cycle pools of violaxanthin + antheraxanthin + zeaxanthin and form more zeaxanthin at peak light exposure every day than individuals of the same plant species growing in low-light environments [21,22]. The regulation of zeaxanthin content thus involves control of (i) xanthophyll cycle pool size, (ii) enzyme-driven xanthophyll cycle interconversions, and (iii) engagement in thermal dissipation facilitated by a pH-sensing protein (PsbS in plants; [23]). When environmental stress is severe and lasts over longer periods, an alternative, pH-independent mechanism becomes active, and zeaxanthin is continuously retained and engaged in thermal dissipation 24 h a day [24]. In most leaves, the key role of the α-carotene-derivative lutein (Figure 1) is one step removed from singlet excited chlorophyll and instead de-excites triplet excited chlorophyll (^3^Chl*; Figure 2; [10]).

Notably, excess absorbed light is not limited to conditions with high light levels; it can also occur when light levels are quite low. When external stressors curb plant growth and the consumption of photosynthate for new growth (and when carbohydrate storage capacity is also exceeded), absorbed light may surpass what can be used in photosynthesis even under low-light conditions. When the sum-total of photosynthetic light utilization (green arrow in Figure 2) and photoprotective thermal dissipation (harmless removal of excess light as thermal energy; blue arrow in Figure 2) does not consume all excitation energy, excitation pressure rises [25] in the chloroplast and triggers ROS-based signaling cascades and further adjustments (red arrow in Figure 2).

**Figure 3:**
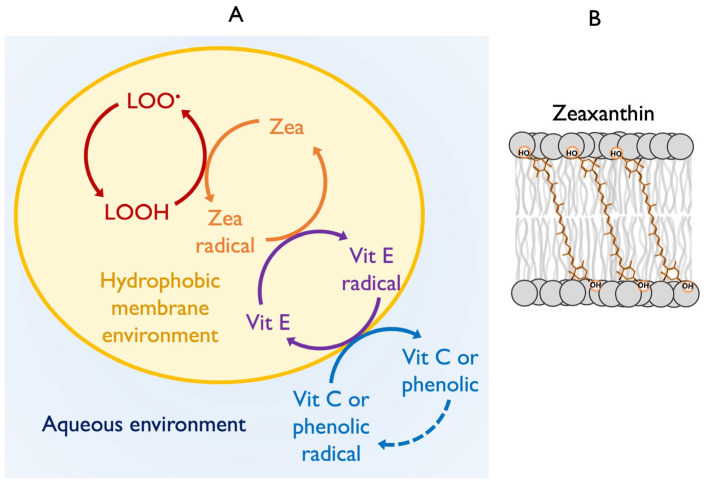
Schematic representation of (**A**) the lipophilic antioxidants operating within a hydrophobic membrane environment (yellow-orange region). Zeaxanthin (Zea), vitamin E (Vit E), and vitamin C (Vit C) or phenolics (the latter two outside of the membrane environment) form an interacting antioxidant network that neutralizes lipid peroxyl radicals (LOO•) in membranes. Each antioxidant can donate electrons to prevent oxidative cascades, with regeneration (via donation of an electron) supported by other antioxidants. (**B**) Schematic depiction of zeaxanthin localization within a biological membrane. Zeaxanthin spans the lipid bilayer, with its polar ends interacting with the polar end groups of membrane phospholipids at the membrane surface.

In addition to its role in regulating light-use efficiency, zeaxanthin also has roles as an antioxidant that can remove singlet excited oxygen once formed and can also counter downstream effects of singlet excited oxygen [1]. Moreover, zeaxanthin can act as a physical stabilizer of the photosynthetic membrane (Figure 1 and Figure 3B; [26]). Lastly, modulation of zeaxanthin level has been shown to alter both the capacity for, and specific targets of, biotic defenses such as herbivores [27] versus systemic acquired resistance [28].

## 4. Carotenoids as Essential Human Micronutrients with Roles in Stress Protection and Gene Regulation

This section notes parallels to carotenoids’ roles in plants for dietary carotenoids as light detectors, protectors against excess light, and regulators for vast gene networks in humans, including systemic effects on the immune system. β-Carotene is provitamin A, the precursor of vitamin A, the latter of which forms an essential part of rhodopsin (the light-absorbing protein) in the human eye [1]. Consequently, vitamin A deficiency can result in blindness. In addition, vitamin A plays a critical role in immune regulation, with severe vitamin A deficiency leading to death due to poor immunity against pathogens [1,29]. Humans require dietary intake of lutein and zeaxanthin as essential micronutrients that cannot be synthesized in the human body. Both zeaxanthin and lutein have important roles in the human eye as well as in the regulation of system-wide immunity responses [2]. In the human eye, zeaxanthin is preferentially deposited into the center of the retina (the macula), where the strongest light is received [1,30]. Conversely, lutein is preferentially deposited into peripheral regions of the eye that serve in low-light vision [1]. Both zeaxanthin and lutein serve in protection of human vision against age-related macular degeneration (blindness; [31]). A particular requirement for zeaxanthin is suggested by the fact that (i) zeaxanthin is enriched relative to lutein between the diet and the retina and (ii) some lutein is converted to the zeaxanthin isomer meso-zeaxanthin that is deposited into the retina’s macular region [1]. Meeting dietary requirements of zeaxanthin from green plant products is difficult due to their typical swift removal of zeaxanthin in low light. Alternative natural sources include, e.g., yellow corn that contains high levels of zeaxanthin and eggs of chickens provided with dietary sources of zeaxanthin [32].

As with β-carotene-derived vitamin A, the immunoregulatory roles of the xanthophyll zeaxanthin serve to oppose chronic inflammation. Chronic inflammation and immune system dysregulation are associated with both poor immunity against infections and with self-attack (autoimmunity) on healthy organs throughout the body [2,33]. Xanthophylls oppose membrane-lipid-oxidation cascades that produce human hormones with immunoregulatory functions and may potentially also have some direct gene-regulatory functions [34] as is the case for vitamin A [35]. The immunoregulatory eicosanoids in animals are equivalent in form and function to plant oxylipins [11] like jasmonates (see Section 6.1), and the effect of xanthophylls on the production of these hormones is another direct parallel between plants and animals. In humans, dietary xanthophylls and antioxidant vitamins assist in opposing production of pro-inflammatory eicosanoids [36].

The next section describes how synergistic interactions between xanthophylls and vitamin E (as well as other antioxidants) effectively counteract membrane lipid oxidation by interacting with downstream products of ROS.

## 5. Roles of Zeaxanthin in Protecting Membrane Integrity and Function

Singlet excited oxygen formed in photosynthesis can oxidize polyunsaturated membrane lipids in several steps, eventually leading to formation of lipid peroxyl radicals (LOO^•^), lipid peroxidation cascades, and membrane destabilization [37]. Zeaxanthin dissolved in the photosynthetic membrane (rather than being bound to chlorophyll-binding complexes) can serve as an antioxidant [38] and, in cooperation with the also membrane-soluble tocopherol (vitamin E), eliminates lipid peroxyl radicals (Figure 3A; [1]). The resulting zeaxanthin radical receives an electron back from tocopherol, and this vitamin E radical is then re-reduced at the membrane–cytosol interface by water-soluble antioxidants like ascorbate (vitamin C) and others (including phenolics). This same membrane-protective antioxidant role of vitamin E and xanthophylls is involved in the above-mentioned opposition to production of lipid-oxidation-based pro-inflammatory human hormones [1].

Lastly, xanthophylls have an additional role in the protection of membrane integrity in organisms ranging from microbes to plants and humans [1,26]. Due to their structure, with large hydrophobic middle portions and hydrophilic groups at both ends, xanthophylls integrate themselves across biological membranes, acting as stabilizing rods (Figure 3B). In this capacity, xanthophylls increase the thermotolerance and overall stability of biological membranes. This role is important in extremophile, highly heat-tolerant microorganisms as well as in plants and humans [1]. Moreover, certain plant species adapted to extreme environments were shown to be capable of zeaxanthin formation even in darkness under conditions of extreme heat or cold [39].

## 6. Specific Functions of Xanthophylls in Modulating Plant Stress Hormone Synthesis

### 6.1. Zeaxanthin, Thermal Dissipation, and Jasmonic Acid

**Figure 4:**
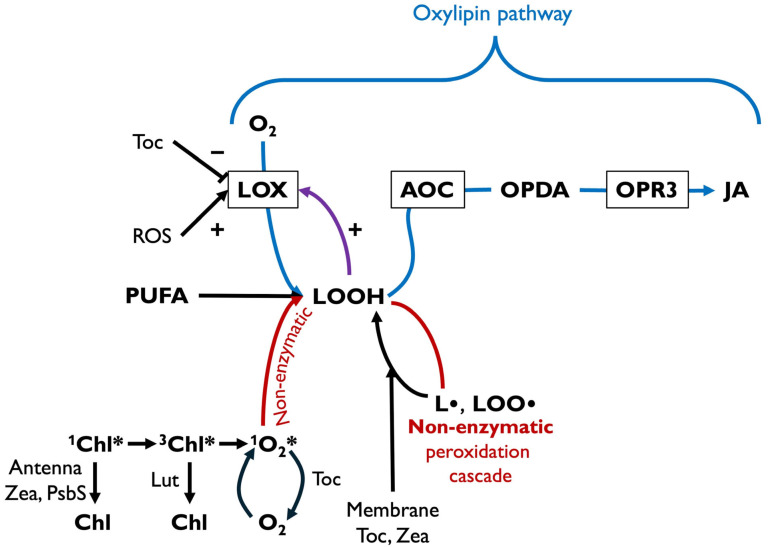
Schematic diagram of the series of steps involved in singlet oxygen formation and non-enzymatic lipid peroxidation, as well as enzymatic lipid peroxidation leading to jasmonic acid (JA) synthesis. Excess singlet excited chlorophyll (^1^Chl*) not utilized in photosynthesis can convert to triplet excited chlorophyll (^3^Chl*), from where energy can be transferred to oxygen, forming singlet excited oxygen (^1^O_2_*). This singlet oxygen forms a lipid hydroperoxide (LOOH), which can presumably contribute to activation of lipoxygenase (LOX), a lipid peroxidase. LOX also produces LOOH, which can further activate LOX, and is also converted to oxylipins like OPDA (via allene oxide cyclase, AOC, in the chloroplast), which is converted to JA by 12-oxophytodienoate reductase (OPR3) in the peroxisome. L•, lipid allylic radical; LOO•, lipid peroxyl radical; Lut, lutein; PsbS, pH-sensitive, thermal dissipation-regulating protein; PUFA, polyunsaturated fatty acid; ROS, reactive oxygen species; Toc, tocopherol; Zea, zeaxanthin; +, stimulation; −, inhibition.

The cascade from excess absorbed light to singlet excited oxygen in the chloroplast can lead to non-enzymatic lipid peroxidation (Figure 4). Specifically, singlet oxygen reacting with a polyunsaturated fatty acid (PUFA) in the chloroplast forms lipid hydroperoxide (LOOH) that gives rise to lipid radicals and lipid peroxyl radicals (Figure 4 [37]). Figure 4 integrates these steps with information described above on ^1^Chl* de-excitation via zeaxanthin and PsbS and ^3^Chl* de-excitation by lutein while also adding singlet oxygen de-excitation by tocopherol [40]. A second, enzymatic peroxidation pathway of membrane lipids, the oxylipin pathway, is facilitated by lipoxygenase (LOX). LOX products are converted via allene oxide cyclase (AOC) to plant oxylipin stress hormones in the chloroplast (Figure 4), such as 12-oxo-phytodienoic acid (OPDA), which is further converted to JA in the peroxisome via 12-oxophytodienoate reductase (OPR3; [11,41]).

The oxylipin pathway is an early warning system, where rising ROS levels in the chloroplasts in stressful environments trigger JA formation. The chloroplast contains high levels of PUFAs, the most oxidation-sensitive membrane phospholipids. These PUFAs are the first to become oxidized by ROS and can thus serve as sentinels for rising ROS levels. The same sentinel function is served by animal PUFAs oxidized to eicosanoid hormones.

LOX produces lipid hydroperoxides, as are also formed by singlet oxygen (Figure 4). Notably, LOOH is also an activator of LOX, thus serving in a feed-forward loop (Figure 4; [42,43]). LOOH formed by singlet oxygen may also serve as an activator of LOX (Figure 4). LOX’s catalytic activity is, furthermore, regulated by ROS and antioxidants (Figure 4). The iron-containing catalytic center of LOX is oxidized to the active form Fe^3+^ by ROS and reduced to the inactive form Fe^2+^ by antioxidants (Figure 4; [11,44]). Moreover, tocopherols act as competitive inhibitors of LOX [45].

In addition to these multiple effects of ROS and antioxidants on LOX activity, expression of the nuclear-encoded genes for both LOX [46] and AOC (Figure 4; [47]) is upregulated by H_2_O_2_ generated in the chloroplast under stress and providing input into signaling networks [48,49]. These events constitute a series of loops of ROS-based signaling that communicate changes in chloroplast redox state (balance between oxidants and antioxidants) to the nucleus and may conversely serve to readjust chloroplast redox state. For example, plants subjected to an increase in growth-light intensity exhibited an initial increase in excitation pressure (as an increased photosystem II reduction state ascertained from the chlorophyll fluorescence parameter 1-q_P_), followed by a subsequent decrease in 1-q_P_ after levels of chloroplast antioxidant metabolites and antioxidant enzymes had increased significantly [22]. When ROS levels in the chloroplast rise under stress, (i) genes in the JA-synthesis pathways are upregulated and (ii) reactive lipid substrate and LOX activity increases. These effects lead to greater JA levels, which then modulate expression of vast gene suites with functions in abiotic defense (against physical stressors such as drought, light, or temperature stress), biotic defense (against pests and some pathogens), and/or plant growth and development (including reproduction and life cycle completion [50,51].

Evidence for a link between zeaxanthin-dependent thermal dissipation (Figure 2) and JA formation was provided by mutant studies. *Arabidopsis thaliana* mutants impaired in the removal of excess ^1^Chl* (deficient in thermal dissipation of excess absorbed light) produced more singlet excited oxygen [52], which is needed for JA synthesis, and also exhibited increased JA levels as well as enhanced JA-dependent phenotypes [11,53]. Remarkably, these mutants simultaneously exhibited enhanced resistance to an insect herbivore [27,54]. This finding is consistent with the role of JA as a plant stress hormone with a particular emphasis on defense against pests. Jasmonic acid is one of two key plant stress hormones with specific, and somewhat antagonistic, roles in biotic defense [55]. Whereas JA targets mainly herbivores and dead-tissue-consuming (necrotrophic) pathogens, salicylic acid (SA) targets mainly live-tissue-consuming (biotrophic) pathogens. Salicylic acid can have a significant negative impact on plant productivity [56], with growth inhibition, accelerated senescence, and decreased seed yield [57,58]. Both SA [59,60] and JA [61,62] thus have multiple functions in plant development [63]. Key steps in both JA and SA biosynthesis occur in the chloroplast [64] and are impacted by chloroplast redox state (see Section 6.3 for details). Moreover, JA and SA show significant reciprocal effects (via what is termed crosstalk) on each other [65]. High levels of JA suppress SA signaling and vice versa [66]. A follow-up study on the above-mentioned *A. thaliana* mutants, deficient in de-excitation of ^1^Chl* via thermal dissipation and overproducing JA, revealed a dramatic suppression of SA-dependent abiotic defense responses [28], which are required for systemic acquired resistance [67].

**Figure 5:**
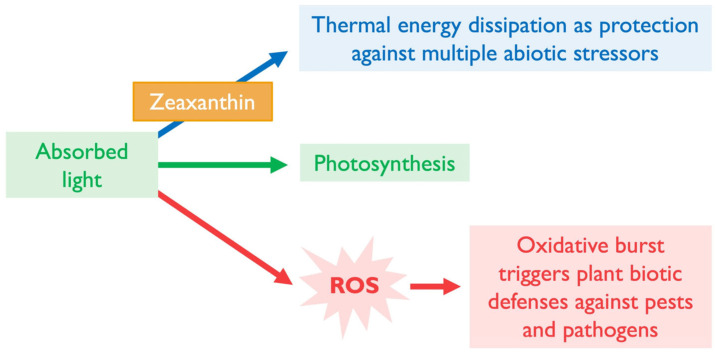
Schematic depiction of the potential trade-off between abiotic and biotic defense. Absorbed light supports photosynthesis but is also required for biotic defense. Under abiotic stress, zeaxanthin safely dissipates excess absorbed light as thermal energy (blue; top arrow). However, some excitation energy must also be used to generate reactive oxygen species (ROS; bottom; red arrow) when an oxidative burst is needed to activate biotic defenses against pests and pathogens.

It is thus clear that there is a potential trade-off between abiotic defense via photoprotective thermal dissipation and biotic defense, which requires what has been termed the “oxidative burst” in the chloroplast needed to activate biotic defenses (Figure 5). The plant must presumably strike a balance between ^1^Chl* de-excitation via thermal dissipation and the production of a robust oxidative burst when needed (Figure 5). In addition, trade-offs between JA- and SA-based defenses and between SA-based biotic defenses and growth might be minimized by a balanced ratio of JA and SA, with each hormone present at moderate levels. Plants need to be nimble while facing multiple different but concurrent stressors in the environment, and more is not always better when it comes to counteracting ROS formation or removing ROS and their products in the chloroplast [68]. Further research is needed to ascertain whether, when, and to what extent thermal dissipation and antioxidation may be downregulated to produce balanced JA- and SA-based defenses. Studying manipulation of plant gene expression by microorganisms may provide further insight, as described in the next section.

### 6.2. Violaxanthin and ABA

The pathogen *Sclerotinia sclerotiorum* increased zeaxanthin formation (by repressing the zeaxanthin-forming enzyme of the xanthophyll cycle, zeaxanthin epoxidase) and decreased violaxanthin levels, which suppressed ABA production and increased stomatal opening as a route of pathogen entry into the leaf [69]. Moreover, increased zeaxanthin formation in the presence of this pathogen was accompanied by increased thermal dissipation (assessed as non-photochemical quenching of chlorophyll fluorescence) and decreased formation of superoxide radical anion (determined using nitroblue tetrazolium; Figure 5; [69]). Conversely, shifting xanthophyll cycle balance from zeaxanthin to violaxanthin may support oxidative-burst generation and retrograde signal transmission through boosting of both JA- and ABA-based stress defenses. These interactions indicate roles of the xanthophyll cycle not only in regulation of light-use efficiency of photosynthesis but also in balancing abiotic and biotic defense, as well as balancing production and signaling of different stress hormones with their own differential trade-off between stress defense and plant productivity [64,70]. This insight further emphasizes that more photoprotection may not always be better [68], that ROS have critical beneficial roles [71,72], and that plants must continuously fine-tune their operation in response to dynamic changes in all factors of their external environment.

### 6.3. Xanthophylls and Salicylic Acid

Whereas salicylic acid is not derived from a carotenoid precursor, its biosynthesis is responsive to redox state. SA can be synthesized by two pathways, both of which start with chorismic acid synthesized in the chloroplast [64]. H_2_O_2_ upregulates gene expression of the key enzyme phenylalanine ammonia-lyase (PAL; [73]) in the PAL pathway and the key enzyme, isochorismate synthase (ICS), in the other pathway (the isochorismate pathway) may also be responsive to H_2_O_2_-based signaling [74]. The latter pathway converts chorismate to isochorismate via ICS and then on to SA in the chloroplast [75]. The PAL pathway of SA synthesis exports chorismate from the chloroplast for conversion to phenylalanine and then to trans-cinnamate (via PAL), followed by several more steps to SA [75]. Suppression of SA-based systemic acquired resistance in a PsbS mutant, which fails to employ thermal dissipation in the de-excitation of ^1^Chl* (Figure 4; [28]), is likely caused by excessive JA levels. The elevated JA production in mutants deficient in zeaxanthin or zeaxanthin and lutein [11] suggests that these xanthophylls may also suppress SA signaling. Together, these findings indicate that manipulation of thermal dissipation and/or singlet oxygen formation—by PsbS, zeaxanthin, and/or lutein—can impact both JA- and SA-based biotic defenses.

## 7. Summary of Multi-Loop Signaling Cascades Between Chloroplast and Nucleus

**Figure 6:**
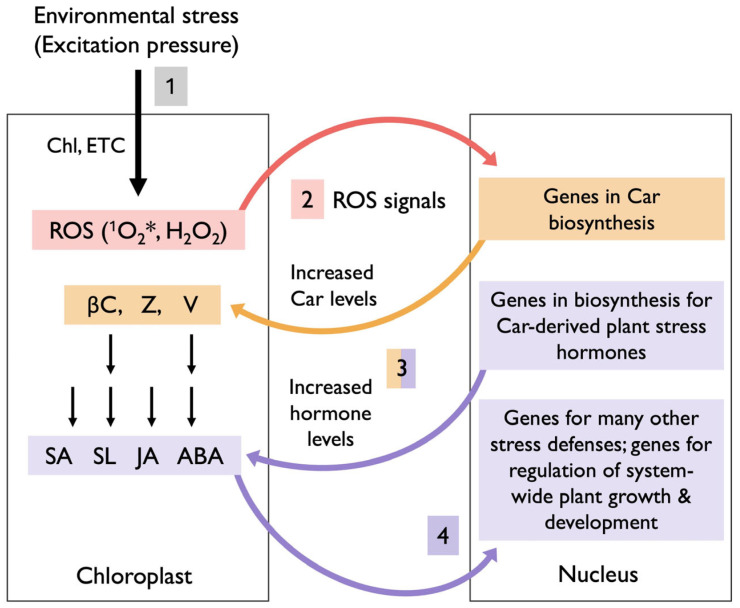
Schematic depiction of a series of steps in signaling cascades between chloroplast and nucleus with multiple reciprocal loops of interaction. The instances where two black arrows are shown in the left box from carotenoids to carotenoid-based hormones depict the conversions between β-carotene (βC) and strigolactones (SL) or between violaxanthin (V) and abscisic acid (ABA), respectively. Single black arrows depict instances of salicylic acid (SA) synthesis that is not directly based on carotenoids or the indirect link between zeaxanthin (Z) and jasmonic acid (JA); Chl, chlorophyll; ETC, photosynthetic electron transport chain; H_2_O_2_, hydrogen peroxide; ^1^O_2_*, singlet excited oxygen; ROS, reactive oxygen species.

Figure 6 presents a summary of the multiple loops for which evidence was described above, with a focus on carotenoids and carotenoid-derived plant stress hormones. These events start with perception of stressors in the external environment via changes in chloroplast redox state in response to changes in excitation pressure (1 in Figure 6). Although excitation pressure is sensed specifically in the chloroplast, it does integrate across the whole plant and multiple factors in the external environment [76,77]. For example, excitation pressure rises when light levels are high enough to cause some oxidative stress, even in a rapidly growing plant that utilizes a lot of absorbed light. As stated above, excitation pressure can also increase when light levels are moderate or low if plant metabolism is depressed (thus lowering utilization of absorbed light in photosynthesis) by environmental stressors, e.g., unfavorable temperatures, limited availability of resources like water or mineral nutrients, or any number of other stressors. All of these environmental stressors presumably trigger a rise in chloroplast ROS levels and result in ROS-based signals to the nucleus (2 in Figure 6), which apparently upregulate genes with (i) roles in the biosynthesis of carotenoids that function in lowering chloroplast ROS levels as well as (ii) genes with roles in the synthesis of plant stress hormones that broadly control stress defenses and other plant stress responses [76,78]. Some products of these nuclear-encoded genes are transported to the chloroplast, where they increase carotenoid levels and hormone production for this type of feedback control (3 in Figure 6). Subsequently, the hormones produced fulfill roles in orchestrating a system-wide plant stress response that involves vast gene networks of nuclear genes, with additional functions in stress defense, as well as system-wide adjustments in plant growth and development (4 in Figure 6).

## 8. Conclusions

The role of chloroplast carotenoids in light-use efficiency and photoprotection and their links to stress hormone biosynthesis confer simultaneous functions to these carotenoids in the chloroplast itself and in system-wide regulation of plant growth, development, and stress defense. An emerging aspect of this larger regulatory function is a potential role of carotenoids in trade-offs between abiotic and biotic defense as well as between the different (JA- vs. SA-based) branches of biotic defense.

These links also offer a new perspective on why most plants absorb considerably more light daily than can be utilized in photosynthesis, while dissipating the excess [2]. This common absorption of excess light may allow for a quick pivot from thermal dissipation (serving in abiotic defense) to production of an oxidative burst (serving in biotic defenses). Such a need for flexibility in dynamic environments offers yet another explanation for why plants continuously adjust xanthophyll cycle pool size and conversion state, as well as antioxidant systems, in response to the environment. This insight suggests that constitutive overexpression of antioxidant systems could have unintended costs and active manipulation in response to stress triggers may be preferable in dynamic natural environments [70]. Lastly, future attention to how plant pathogens, and possibly microbial symbionts, tune these systems may provide guidance for developing plant lines with improved resilience and productivity in complex, changing, and challenging environments.

## References

[1] Demmig-Adams B., Polutchko S.K., Adams W.W. (2022). Structure-Function-Environment Relationship of the Isomers Zeaxanthin and Lutein. Photochem.

[2] Demmig-Adams B., Stewart J.J., López-Pozo M., Polutchko S.K., Adams W.W. (2020). Zeaxanthin, a Molecule for Photoprotection in Many Different Environments. Molecules.

[3] Foyer C.H., Kunert K. (2024). The Ascorbate–Glutathione Cycle Coming of Age. J. Exp. Bot..

[4] Niyogi K.K. (1999). Photoprotection Revisited: Genetic and Molecular Approaches. Annu. Rev. Plant Biol..

[5] Uarrota V.G., Stefen D.L.V., Leolato L.S., Gindri D.M., Nerling D., Gupta D., Palma J., Corpas F. (2018). Revisiting Carotenoids and Their Role in Plant Stress Responses: From Biosynthesis to Plant Signaling Mechanisms during Stress. Antioxidants and Antioxidant Enzymes in Higher Plants.

[6] Zhao B., Liu Q., Wang B., Yuan F. (2021). Roles of Phytohormones and Their Signaling Pathways in Leaf Development and Stress Responses. J. Agric. Food Chem..

[7] Brewer P.B., Koltai H., Beveridge C.A. (2013). Diverse Roles of Strigolactones in Plant Development. Mol. Plant.

[8] Bhoi A., Yadu B., Chandra J., Keshavkant S. (2021). Contribution of Strigolactone in Plant Physiology, Hormonal Interaction and Abiotic Stresses. Planta.

[9] Özbilen A., Sezer F., Taşkin K.M. (2024). Identification and Expression of Strigolactone Biosynthesis and Signaling Genes and the in Vitro Effects of Strigolactones in Olive (*Olea europaea* L.). Plant Direct.

[10] Dall’Osto L., Lico C., Alric J., Giuliano G., Havaux M., Bassi R. (2006). Lutein Is Needed for Efficient Chlorophyll Triplet Quenching in the Major LHCII Antenna Complex of Higher Plants and Effective Photoprotection in Vivo under Strong Light. BMC Plant Biol..

[11] Demmig-Adams B., Cohu C.M., Amiard V., Van Zadelhoff G., Veldink G.A., Muller O., Adams W.W. (2013). Emerging Trade—offs—Impact of Photoprotectants (PsbS, Xanthophylls, and Vitamin E) on Oxylipins as Regulators of Development and Defense. New Phytol..

[12] North H.M., Almeida A.D., Boutin J., Frey A., To A., Botran L., Sotta B., Marion-Poll A. (2007). The Arabidopsis ABA-deficient Mutant *aba4* Demonstrates That the Major Route for Stress-induced ABA Accumulation Is via Neoxanthin Isomers. Plant J..

[13] Huang Y., Guo Y., Liu Y., Zhang F., Wang Z., Wang H., Wang F., Li D., Mao D., Luan S. (2018). 9-Cis-Epoxycarotenoid Dioxygenase 3 Regulates Plant Growth and Enhances Multi-Abiotic Stress Tolerance in Rice. Front. Plant Sci..

[14] Slovik S., Daeter W., Hartung W. (1995). Compartmental Redistribution and Long-Distance Transport of Abscisic Acid (ABA) in Plants as Influenced by Environmental Changes in the Rhizosphere—A Biomathematical Model. J. Exp. Bot..

[15] Lee T.-Y., Lam L., Patel-Tupper D., Roy P.P., Ma S.A., Lam H.E., Lucas-DeMott A., Karavolias N.G., Iwai M., Niyogi K.K. (2024). Chlorophyll to Zeaxanthin Energy Transfer in Nonphotochemical Quenching: An Exciton Annihilation-Free Transient Absorption Study. Proc. Natl. Acad. Sci. USA.

[16] Krieger-Liszkay A. (2005). Singlet Oxygen Production in Photosynthesis. J. Exp. Bot..

[17] Kozuleva M.A., Ivanov B.N. (2023). Superoxide Anion Radical Generation in Photosynthetic Electron Transport Chain. Biochem. Mosc..

[18] Pospíšil P. (2009). Production of Reactive Oxygen Species by Photosystem II. Biochim. Biophys. Acta BBA-Bioenerg..

[19] Murchie E.H., Ruban A.V. (2020). Dynamic Non-photochemical Quenching in Plants: From Molecular Mechanism to Productivity. Plant J..

[20] Baroli I., Niyogi K.K. (2000). Molecular Genetics of Xanthophyll–Dependent Photoprotection in Green Algae and Plants. Philos. Trans. R. Soc. Lond. B Biol. Sci..

[21] Demmig-Adams B., Adams W.W. (1992). Carotenoid Composition in Sun and Shade Leaves of Plants with Different Life Forms. Plant Cell Environ..

[22] Logan B.A., Demmig-Adams B., Adams W.W., Grace S.C. (1998). Antioxidants and Xanthophyll Cycle-Dependent Energy Dissipation in *Cucurbita pepo* L. and *Vinca major* L. Acclimated to Four Growth PPFDs in the Field. J. Exp. Bot..

[23] Li X.-P., Gilmore A.M., Caffarri S., Bassi R., Golan T., Kramer D., Niyogi K.K. (2004). Regulation of Photosynthetic Light Harvesting Involves Intrathylakoid Lumen pH Sensing by the PsbS Protein. J. Biol. Chem..

[24] Demmig-Adams B., Adams W.W. (2006). Photoprotection in an Ecological Context: The Remarkable Complexity of Thermal Energy Dissipation. New Phytol..

[25] Hüner N.P., Bode R., Dahal K., Hollis L., Rosso D., Krol M., Ivanov A.G. (2012). Chloroplast Redox Imbalance Governs Phenotypic Plasticity: The “Grand Design of Photosynthesis” Revisited. Front. Plant Sci..

[26] Havaux M. (1998). Carotenoids as Membrane Stabilizers in Chloroplasts. Trends Plant Sci..

[27] Johansson Jänkänpää H., Frenkel M., Zulfugarov I., Reichelt M., Krieger-Liszkay A., Mishra Y., Gershenzon J., Moen J., Lee C.-H., Jansson S. (2013). Non-Photochemical Quenching Capacity in *Arabidopsis thaliana* Affects Herbivore Behaviour. PLoS ONE.

[28] Göhre V., Jones A.M.E., Sklenář J., Robatzek S., Weber A.P.M. (2012). Molecular Crosstalk Between PAMP-Triggered Immunity and Photosynthesis. Mol. Plant-Microbe Interact..

[29] Semba R.D. (1994). Vitamin A, Immunity, and Infection. Clin. Infect. Dis..

[30] Kumar P., Banik S.P., Ohia S.E., Moriyama H., Chakraborty S., Wang C.-K., Song Y.S., Goel A., Bagchi M., Bagchi D. (2024). Current Insights on the Photoprotective Mechanism of the Macular Carotenoids, Lutein and Zeaxanthin: Safety, Efficacy and Bio-Delivery. J. Am. Nutr. Assoc..

[31] Mrowicka M., Mrowicki J., Kucharska E., Majsterek I. (2022). Lutein and Zeaxanthin and Their Roles in Age-Related Macular Degeneration—Neurodegenerative Disease. Nutrients.

[32] Perry A., Rasmussen H., Johnson E.J. (2009). Xanthophyll (Lutein, Zeaxanthin) Content in Fruits, Vegetables and Corn and Egg Products. J. Food Compos. Anal..

[33] Kanterman J., Sade-Feldman M., Baniyash M. (2012). New Insights into Chronic Inflammation-Induced Immunosuppression. Semin. Cancer Biol..

[34] Eroglu A., Harrison E.H. (2013). Carotenoid Metabolism in Mammals, Including Man: Formation, Occurrence, and Function of Apocarotenoids. J. Lipid Res..

[35] McGrane M.M. (2007). Vitamin A Regulation of Gene Expression: Molecular Mechanism of a Prototype Gene. J. Nutr. Biochem..

[36] Polutchko S.K., Glime G.N.E., Demmig-Adams B. (2021). Synergistic Action of Membrane-Bound and Water-Soluble Antioxidants in Neuroprotection. Molecules.

[37] Kumar A., Prasad A., Sedlářová M., Ksas B., Havaux M., Pospíšil P. (2020). Interplay between Antioxidants in Response to Photooxidative Stress in Arabidopsis. Free Radic. Biol. Med..

[38] Havaux M., García-Plazaola J.I., Demmig-Adams B., Garab G., Adams W.W., III Govindjee (2014). Beyond Non-Photochemical Fluorescence Quenching: The Overlapping Antioxidant Functions of Zeaxanthin and Tocopherols. Non-Photochemical Quenching and Energy Dissipation in Plants, Algae and Cyanobacteria.

[39] Fernández-Marín B., Roach T., Verhoeven A., García-Plazaola J.I. (2021). Shedding Light on the Dark Side of Xanthophyll Cycles. New Phytol..

[40] Triantaphylidès C., Havaux M. (2009). Singlet Oxygen in Plants: Production, Detoxification and Signaling. Trends Plant Sci..

[41] Schaller F., Biesgen C., Müssig C., Altmann T., Weiler E.W. (2000). 12-Oxophytodienoate Reductase 3 (OPR3) Is the Isoenzyme Involved in Jasmonate Biosynthesis. Planta.

[42] Jones G.D., Russell L., Darley-Usmar V.M., Stone D., Wilson M.T. (1996). Role of Lipid Hydroperoxides in the Activation of 15-Lipoxygenase. Biochemistry.

[43] Zheng Y., Brash A.R. (2010). On the Role of Molecular Oxygen in Lipoxygenase Activation. J. Biol. Chem..

[44] Maccarrone M., Lorenzon T., Guerrieri P., Agrò A.F. (1999). Resveratrol Prevents Apoptosis in K562 Cells by Inhibiting Lipoxygenase and Cyclooxygenase Activity. Eur. J. Biochem..

[45] Park N.-Y., Im S., Jiang Q. (2022). Different Forms of Vitamin E and Metabolite 13′-Carboxychromanols Inhibit Cyclooxygenase-1 and Its Catalyzed Thromboxane in Platelets, and Tocotrienols and 13′-Carboxychromanols Are Competitive Inhibitors of 5-Lipoxygenase. J. Nutr. Biochem..

[46] Maccarrone M., Van Zadelhoff G., Veldink G.A., Vliegenthart J.F.G., Finazzi-Agrò A. (2000). Early Activation of Lipoxygenase in Lentil (*Lens culinaris*) Root Protoplasts by Oxidative Stress Induces Programmed Cell Death. Eur. J. Biochem..

[47] Agrawal G. (2003). Cloning of Novel Rice Allene Oxide Cyclase (OsAOC): mRNA Expression and Comparative Analysis with Allene Oxide Synthase (OsAOS) Gene Provides Insight into the Transcriptional Regulation of Octadecanoid Pathway Biosynthetic Genes in Rice. Plant Sci..

[48] Breeze E., Mullineaux P.M. (2022). The Passage of H_2_O_2_ from Chloroplasts to Their Associated Nucleus during Retrograde Signalling: Reflections on the Role of the Nuclear Envelope. Plants.

[49] Foyer C.H., Lopez-Delgado H., Dat J.F., Scott I.M. (1997). Hydrogen Peroxide- and Glutathione-associated Mechanisms of Acclimatory Stress Tolerance and Signalling. Physiol. Plant..

[50] Creelman R.A., Mullet J.E. (1995). Jasmonic Acid Distribution and Action in Plants: Regulation during Development and Response to Biotic and Abiotic Stress. Proc. Natl. Acad. Sci. USA.

[51] Santino A., Taurino M., De Domenico S., Bonsegna S., Poltronieri P., Pastor V., Flors V. (2013). Jasmonate Signaling in Plant Development and Defense Response to Multiple (a)Biotic Stresses. Plant Cell Rep..

[52] Roach T., Krieger-Liszkay A. (2012). The Role of the PsbS Protein in the Protection of Photosystems I and II against High Light in *Arabidopsis thaliana*. Biochim. Biophys. Acta BBA—Bioenerg..

[53] Külheim C., Jansson S. (2005). What Leads to Reduced Fitness in Non-photochemical Quenching Mutants?. Physiol. Plant..

[54] Frenkel M., Külheim C., Jänkänpää H.J., Skogström O., Dall’Osto L., Ågren J., Bassi R., Moritz T., Moen J., Jansson S. (2009). Improper Excess Light Energy Dissipation in *Arabidopsis* Results in a Metabolic Reprogramming. BMC Plant Biol..

[55] Glazebrook J. (2005). Contrasting Mechanisms of Defense Against Biotrophic and Necrotrophic Pathogens. Annu. Rev. Phytopathol..

[56] Pokotylo I., Hodges M., Kravets V., Ruelland E. (2022). A Ménage à Trois: Salicylic Acid, Growth Inhibition, and Immunity. Trends Plant Sci..

[57] Toçilla S. (2024). In the Spotlight: The Plant Growth-Defence Dilemma: A Hormonal Balancing Act. Physiol. Plant..

[58] Yang L. (2024). You Can Have Your Cake and Eat It Too: Ectopic Expression of Cold-Regulated Genes Reshapes the Salicylic Acid–Mediated Growth-Defense Tradeoff. Plant Cell.

[59] Hu Y., Zhi L., Li P., Hancock J.T., Hu X. (2022). The Role of Salicylic Acid Signal in Plant Growth, Development and Abiotic Stress. Phyton.

[60] Rivas-San Vicente M., Plasencia J. (2011). Salicylic Acid beyond Defence: Its Role in Plant Growth and Development. J. Exp. Bot..

[61] Jang G., Yoon Y., Choi Y.D. (2020). Crosstalk with Jasmonic Acid Integrates Multiple Responses in Plant Development. Int. J. Mol. Sci..

[62] Liu H., Timko M.P. (2021). Jasmonic Acid Signaling and Molecular Crosstalk with Other Phytohormones. Int. J. Mol. Sci..

[63] Santisree P., Jalli L.C.L., Bhatnagar-Mathur P., Sharma K.K., Aryadeep Roychoudhury A., Kumar Tripathi D. (2020). Emerging Roles of Salicylic Acid and Jasmonates in Plant Abiotic Stress Responses. Protective Chemical Agents in the Amelioration of Plant Abiotic Stress.

[64] Lu Y., Yao J. (2018). Chloroplasts at the Crossroad of Photosynthesis, Pathogen Infection and Plant Defense. Int. J. Mol. Sci..

[65] Reymond P., Farmer E.E. (1998). Jasmonate and Salicylate as Global Signals for Defense Gene Expression. Curr. Opin. Plant Biol..

[66] Li N., Han X., Feng D., Yuan D., Huang L.-J. (2019). Signaling Crosstalk between Salicylic Acid and Ethylene/Jasmonate in Plant Defense: Do We Understand What They Are Whispering?. Int. J. Mol. Sci..

[67] Klessig D.F., Choi H.W., Dempsey D.A. (2018). Systemic Acquired Resistance and Salicylic Acid: Past, Present, and Future. Mol. Plant-Microbe Interact..

[68] Demmig-Adams B., Stewart J.J., Adams W.W. (2019). Less Photoprotection Can Be Good in Some Genetic and Environmental Contexts. Biochem. J..

[69] Zhou J., Zeng L., Liu J., Xing D. (2015). Manipulation of the Xanthophyll Cycle Increases Plant Susceptibility to *Sclerotinia sclerotiorum*. PLoS Pathog..

[70] Kerchev P.I., Van Breusegem F. (2022). Improving Oxidative Stress Resilience in Plants. Plant J..

[71] Fichman Y., Rowland L., Oliver M.J., Mittler R. (2023). ROS Are Evolutionary Conserved Cell-to-Cell Stress Signals. Proc. Natl. Acad. Sci. USA.

[72] Mittler R. (2017). ROS Are Good. Trends Plant Sci..

[73] Jeong M.-J., Choi B.S., Bae D.W., Shin S.C., Park S.U., Lim H.-S., Kim J., Kim J.B., Cho B.-K., Bae H. (2012). Differential Expression of Kenaf Phenylalanine Ammonia-Lyase (PAL) Ortholog during Developmental Stages and in Response to Abiotic Stresses. Plant Omics.

[74] Chaouch S., Queval G., Vanderauwera S., Mhamdi A., Vandorpe M., Langlois-Meurinne M., Van Breusegem F., Saindrenan P., Noctor G. (2010). Peroxisomal Hydrogen Peroxide Is Coupled to Biotic Defense Responses by ISOCHORISMATE SYNTHASE1 in a Daylength-Related Manner. Plant Physiol..

[75] Lefevere H., Bauters L., Gheysen G. (2020). Salicylic Acid Biosynthesis in Plants. Front. Plant Sci..

[76] Foyer C.H., Noctor G. (2013). Redox Signaling in Plants. Antioxid. Redox Signal..

[77] Mittler R., Jones D.P. (2024). The Redox Code of Plants. Plant Cell Environ..

[78] Noctor G., Reichheld J.-P., Foyer C.H. (2018). ROS-Related Redox Regulation and Signaling in Plants. Semin. Cell Dev. Biol..

